# Fiber Bragg Grating Sensors for Millimetric-Scale Temperature Monitoring of Cardiac Tissue Undergoing Radiofrequency Ablation: A Feasibility Assessment

**DOI:** 10.3390/s20226490

**Published:** 2020-11-13

**Authors:** Martina Zaltieri, Greta Allegretti, Carlo Massaroni, Emiliano Schena, Filippo Maria Cauti

**Affiliations:** 1 Department of Engineering, Università Campus Bio-Medico di Roma, Via Alvaro del Portillo, 00128 Rome, Italy; m.zaltieri@unicampus.it (M.Z.); c.massaroni@unicampus.it (C.M.); 2 Abbott Medical, Viale Thomas Alva Edison, 20099 Sesto San Giovanni, Italy; greta.allegretti@abbott.com; 3 Arrhythmology Unit, Cardiology Division, S. Giovanni Calibita Hospital, Isola Tiberina, 00186 Rome, Italy; filippocauti@hotmail.it

**Keywords:** temperature measurements, fiber Bragg grating sensors, fiber optic sensors, myocardial radiofrequency ablation, radiofrequency ablation

## Abstract

Radiofrequency ablation (RFA) is the most widely used technique for the treatment of cardiac arrhythmias. A variety of factors, such as the electrode tip shape, the force exerted on the tissue by the catheter and the delivered power, combine to determine the temperature distribution, and as consequence, the lesion shape and size. In this context, being able to know the temperature reached in the myocardium during the RFA can be helpful for predicting the lesion dimensions to prevent the occurrence of undesired tissue damage. The catheters used so far in such procedures provide single-point temperature measurements within the probe (by means of embedded thermocouples or thermistors), so no information regarding the temperature changes occurring in myocardial tissues can be retrieved. The aim of this study was to assess the feasibility of fiber Bragg grating sensors (FBGs) to perform multi-point and millimetric-scale temperature measurements within myocardium subjected to RFA. The assessment has been performed on ex vivo porcine myocardium specimens undergoing RFA. Data show the feasibility of the proposed solution in providing spatial temperature distribution within the myocardial tissue during the entire RFA. These high-resolved measurements may allow reconstructing the temperature distribution in the tissue. This study lays the foundations for the implementation of 3D thermal maps to investigate how the supplied power, treatment time, force of contact and irrigation flow of the catheter influence the thermal effects within the tissue.

## 1. Introduction

Cardiac arrhythmias represent a relevant epidemiological problem which implies serious cardiovascular complications, and as consequence, important healthcare expenses [1].

Among other minimally invasive thermal treatments (MITT), antenna-mediated radiofrequency ablation (RFA) has prevailed as leading process to treat such diseases [2,3]. The aim of RFA is to induce irreversible loss of cellular excitability through local hyperthermia by delivering a certain amount of energy in specific portions of the myocardial tissue by means of a RF antenna. In order to ensure unrecoverable cell destruction, temperatures of at least 50 °C (i.e., lethal isotherm [4]) must be reached. On the contrary, temperatures far above this value should not be achieved to avoid harmful complications such as steam popping, tissue perforations and formations of hematic and tissue coagulums upon the antenna tip [5,6,7,8]. Moreover, the electrode tip shape, the force exerted on the myocardium by the catheter, the tip irrigation and the delivered power are the main factors that combine in determining the temperature distribution within the treated tissue, and as consequence, the lesion shape and size [9,10,11]. Therefore, since the tissue heating is significantly related to the success or the failing of RFA treatments, being able to reconstruct the spatial and temporal evolution of the temperatures reached within the tissue can be crucial to ensure a successful outcome and may also help to predict the dimensions of the thermally damaged areas.

To date, several methods have been used to investigate the temperature within the myocardium subjected to RFA, but in clinical practice the temperature is measured in the RF antennas only by the embedded thermocouples and thermistors [12,13,14,15,16]. Nevertheless, the use of such approach returns single-point temperature values only: in fact, measurements are performed uniquely at the antenna tip placed on the myocardial surface. Thus, no thermal information regarding the underlying tissues is provided. Moreover, the convective heat loss caused by continuous interactions between the antenna tip and the surrounding body environment (i.e., blood and tissues), the cooling system of the antenna delivering saline solution and tip-to-tissue contact can return lower temperature values than the actual tissue temperature [3].

Some contactless solutions based on magnetic resonance imaging (MRI) techniques have been proposed for temperature mapping [17,18]. Despite the advantages brought by the non-invasiveness of the approach, their utilization in a real clinical scenario is discouraged by several factors (e.g., the image artifacts caused by cardiac motion, the extremely high costs of the procedure and the requirement of using MRI-compatible surgical tools).

Still on the topic of contactless techniques, ultrasound-based solutions offer a less expensive alternative that permit to obtain bi-dimensional real-time temperature mapping [19] exploiting the linear relationship between temperature variation and frequency variation of the retro-reflected signal [20]. Nevertheless, such techniques are ineffective for RF procedures in which temperatures greater than 50 °C are required. In fact, at certain temperatures tissues undergo state changes that might modify the ultrasound backscattered signal, providing unrealistic thermal measurements [20,21]. That makes the technology unsuitable for the clinical practice.

A further contactless technique is based on the use of computed tomography (CT) images (i.e., CT-thermometry). Although this technique has already been used for monitoring temperature during hyperthermal treatments in surgical oncology, studies are limited to animal models, and CT-thermometry does not seem feasible for this application considering the presence of motion image artifacts and the lack of high temporal and spatial resolution [22].

In this context, the use of arrays embedding fiber Bragg grating (FBG) sensors allow providing spatial temperature reconstructions in myocardial tissues undergoing RFA by performing minimally-invasive, high-resolved and distributed temperature measurements. Indeed, FBGs’ features, such as small dimension, multiplexing capability and immunity to electromagnetic fields, make these sensors suitable for this purpose [23,24].

This is the first study investigating the feasibility of a new FBG-based approach for high-resolved temperature mapping in myocardium. The assessment has been carried out on ex vivo porcine myocardium specimens undergoing RFA at two different power settings. Temperature trends were investigated in several measurement sites and the sizes of the lesions produced were evaluated.

## 2. Fiber Bragg Grating Sensors: Working Principle and Temperature Calibration

In the following paragraphs, the working principle of the FBG sensors and their temperature calibration are described.

### 2.1. Fiber Bragg Grating’s Working Principle

To perform high-resolved temperature measurements within myocardial tissue specimens undergoing RFA, two nominally identical optical fibers (FiSens GmbH, Braunschweig, Germany), each one embedding 7 FBG sensors, were employed. The FBGs displayed the following specifications: 1 mm of length with 2 mm of in between distance one from another, acrylate coating, reflectivity value >20%, and FWHM value <2 nm. The total sensitive length turned out to be 19 mm for each fiber. Moreover, each sensor was set at a different working wavelength, called Bragg wavelength (λ_B_), ranging from 1500 to 1600 nm. Such FBG arrays were produced by modulating the periodic refractive index of the fiber core via inscribing small portions of cladding by means of an intense UV light. Exploiting the point-by-point laser procedure described in [25] which ensures high precision gratings inscribing, it was possible to realize sensors of such small dimensions (i.e., 1 mm).

**Figure 1:**
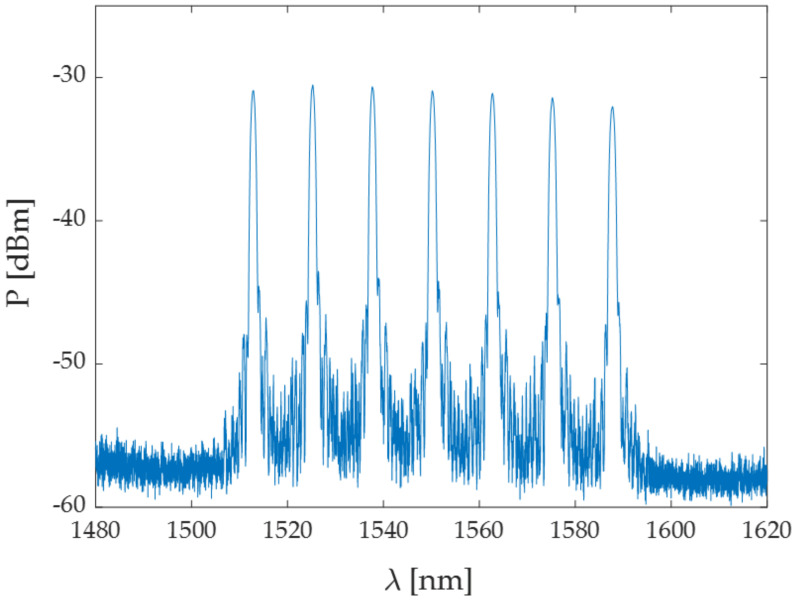
Optical spectrum shown by one of the two nominally identical arrays. The seven peaks corresponding to the λ_B_ of each fiber Bragg grating (FBG) are clearly distinguishable.

A fiber optic interrogator was used to illuminate the FBGs with a full spectrum light source. Each FBG acts like a passband filter, transmitting most of the light and reflecting back as output a narrow spectrum centered around its λ_B_ [26]. The spectrum of one of the two nominally identical arrays is reported in Figure 1 as an example.

As indicated in the following equation [27], the λ_B_ is function of the core effective refractive index (η_eff_) and the grating period (Λ):

λ_B_ = 2 · η_eff_ · Λ (1)

Temperature variations (ΔT), and deformations (ε), deeply influence η_eff_ and Λ so producing a λ_B_ shift (Δλ_B_). When the strain is negligible, Δλ_B_ can be considered only related to ΔT.

### 2.2. Temperature Calibration of the FBG Sensors

**Figure 2:**
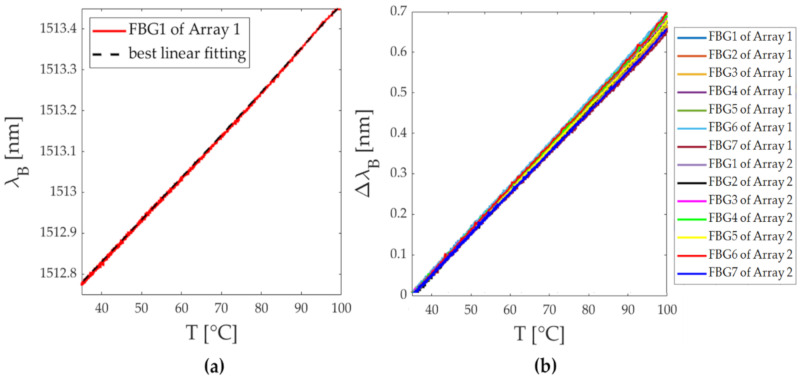
(**a**) The thermal response of the FBG 1 embedded in Array 1 (red line) and the best linear fitting (black dash line); (**b**) the temperature calibration curves of the fourteen FBGs embedded in Array 1 and Array 2.

The response of the sensors to ΔT was evaluated to calculate their thermal sensitivity values (S_T_). The two arrays (Array 1 and Array 2) were placed into a laboratory oven (PN120 Carbolite^®^) together with a thermistor (EL-USB-TC-LCD, EasyLog, Lascar Technology, Whiteparish, UK) and subjected to a temperature variation of 95 °C (i.e., from 35 °C to 100 °C of temperature range). The chosen temperature range includes the temperatures to which the arrays were exposed during the experimental tests. The outputs of the FBGs were acquired by a fiber optical interrogator (sm125, Micron Optics Inc., Atlanta, GA, USA) with a sampling frequency of 1 Hz, while the thermistor recorded temperature values at 0.2 Hz. The temperature calibration curve (i.e., λ_B_ vs. T) was obtained for each sensor. In Figure 2a the calibration curve of the FBG 1 embedded in Array 1 is reported as an example. In Figure 2b the calibration curves of all the FBGs belonging to Arrays 1 and 2 are shown; unlike the previous case, the Δλ_B_ vs. T were shown since the sensors were set at different λ_B_, and as a consequence the λ_B_ variations would not have been appreciated if reported together.

The values of the S_T_ were calculated as the slope of the best fitting line. The S_T_ values related to the fourteen FBGs are reported in Table 1. As is visible, the obtained S_T_ values are clearly comparable to the ones declared by the manufacturer (i.e., 0.01 nm·°C^−1^).

## 3. Radiofrequency Ablation of Ex Vivo Porcine Myocardium

Multi-point temperature measurements on ex vivo porcine myocardium subjected to RFA were performed. In the following paragraphs, the experimental setup and the sensors’ positions in the tissue specimen are reported.

### 3.1. Experimental Setup

**Figure 3:**
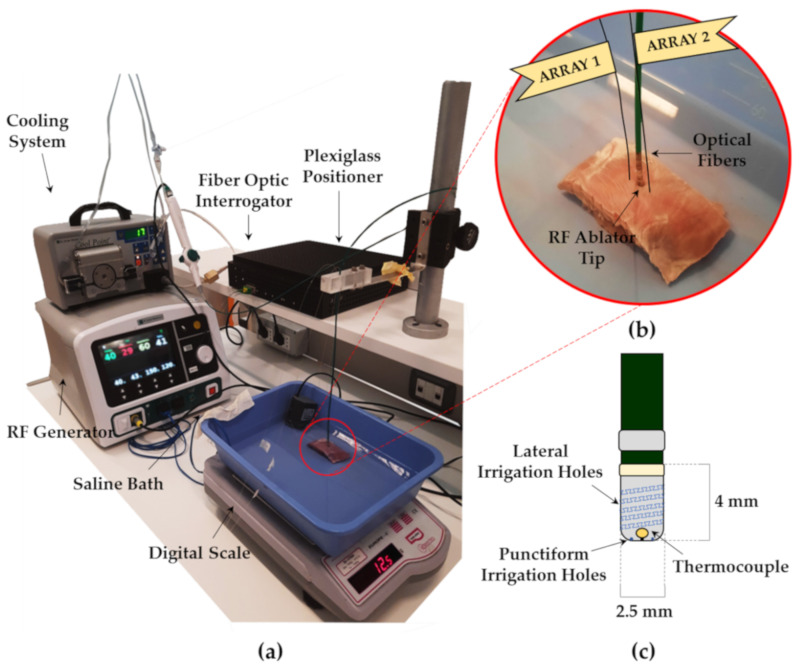
The experimental setup used to perform radiofrequency ablation (RFA) of the myocardial tissue. (**a**) Photo of the setup; (**b**) a zoom-in on the myocardial specimen showing the optical fibers and the RF ablator placement; (**c**) a graphic representation of the RF catheter showing the antenna tip, the thermocouple (located at 0.3 mm from the distal tip), and the lateral and punctiform irrigation holes.

Two sections of myocardial tissue were subjected to RFA for 60 s of treatment time at power settings of 50 W (Trial 1) and 60 W (Trial 2), respectively. Swine hearts were provided from the local slaughterhouse. The cardiac tissue was excised and two specimens of myocardium whose dimensions were about 2 × 4 × 3 cm were obtained; then each tissue sample was placed into a saline bath. To simulate the human body’s physiological conditions, the saline bath was maintained at the temperature of about 37 °C and a water pump providing a flowrate of 5 mL·min^−1^ was used. A RF Generator (Generatore Ampere, Abbott Medical, MN, USA), connected to a RF antenna (FlexAbility^™^ Ablation Catheter Sensor Enabled^™^, Abbott Medical, MN, USA), whose tip diameter was 2.5 mm, and a cooling system (Cool Point™, Abbott Medical, MN, USA) dispensing 17 mL·min^−1^ of saline solution were employed. The tip of the RF antenna was positioned on the surface of the myocardial tissue and held still by means of a perforated plexiglass positioning structure. Two optical fibers, housing an array of 7 FBGs each, were carefully inserted into the specimen with the help of two stiff needles—20 Gauge calipered. The two optical fibers were introduced into the holes of the plexiglass positioner and placed parallel to the RF antenna at different distances. Being careful to leave all the FBG sensors inside the tissue, the needles were then removed and the fibers were maintained by the positioner. The experimental setup and the fiber positioning are shown in Figure 3.

To replicate the conditions of clinical practice, slight pressure was then exerted on the RF antenna to impart on the tissue a force of about 12 gf (i.e., 0.117 N), measured by a digital scale placed under the saline bath. An optical fiber interrogator (Optical Sensing Interrogator, si255 based on Hyperion Platform, Micron Optics, Atlanta, GA, USA) was used to collect the data provided by the FBG sensors at a sampling rate of 1000 Hz. At the end of the RFA treatments, the myocardial specimens were sliced in order to evaluate the dimensions of the produced lesions.

### 3.2. Sensors and Antenna Positioning into the Tissue during Radiofrequency Ablation

**Figure 4:**
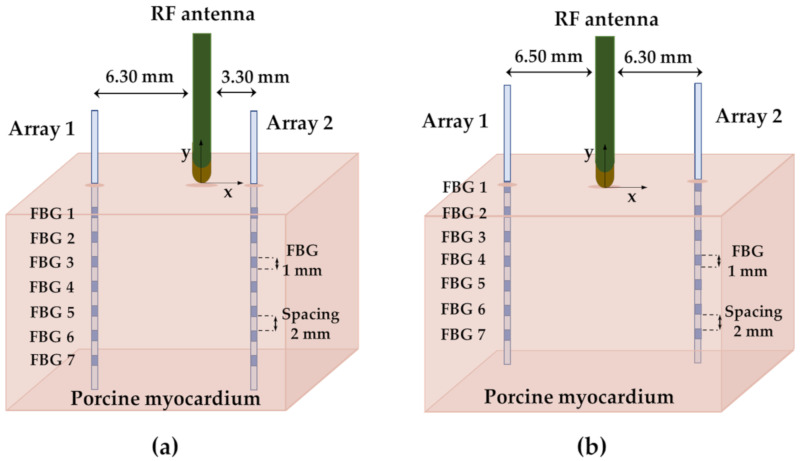
Schematic of the FBG sensors and RF antenna positioning in the myocardial tissue during the RF ablation process: (**a**) setting for the ablation performed at 50 W (Trial 1); (**b**) setting for the ablation performed at 60 W (Trial 2).

Two optical fibers embedding 7 FBGs each and a RF antenna were positioned respectively into and upon the tissue specimen during both the experiments performed at 50 and 60 W. The RF catheter was placed orthogonally to the skin and in contact with the surface, while the Arrays 1 and 2 were inserted into the tissue in parallel to the catheter to its right and left, respectively (Figure 4). The optical fibers were inserted into the myocardial tissue by means of two 20 Gauge calipered needles. Firstly, the needles were passed through two holes of the plexiglass positioner and introduced into the organ at specific distances from the antenna tip. Each array was inserted into the needles that provided a guidance path for their placement. The FBGs positioning along the specimen was ensured through a reference mark. The needles were then carefully extracted from the myocardium and removed from the positioner, while paying close attention not to alter the placement of the FBGs. The positioning of the arrays (distance of each fiber from the RF antenna) was chosen to ensure the presence of at least two sensors inside the lesion. In fact, as reported in [11], the expected diameter of a lesion produced on the myocardium through RF ablation with 50 W of delivered power and 60 s of treatment time is 10.7 ± 1.1 mm. Moreover, in both the arrays, the FBGs were named with a progressive number, starting from FBG 1 (which was the closest to the surface) up to FBG 7 (which was the one placed deeper into the tissue). In Trial 1 and Trial 2, the FBGs 1 of both the arrays were placed at different depths in order to evaluate the influence of cooling effect in relation to the proximity to the surface.

The spatial configuration referring to the experiment performed at 50 W is shown in Figure 4a. Arrays 1 and 2 were 6.30 and 3.30 mm distant from the RF antenna, respectively, while both the FBGs 1 were placed at about 2 mm under the tissue surface. Moreover, the spatial configuration referring to the experiment performed at 60 W is shown in Figure 4b. The Arrays 1 and 2 were 6.50 and 6.30 mm distant from the RF antenna, respectively, while both the FBGs 1 were placed directly below the surface of the tissue.

Considering the tip of the antenna as the origin of a x-y plane for both the settings of Trial 1 and Trial 2, the coordinates of each FBG belonging to Array 1 and Array 2 are reported in Table 2.

## 4. Results

In Trial 1 and Trial 2, two different porcine myocardial specimens were subjected to RFA. Temperature trends were investigated, and the sizes of the lesions produced were evaluated. In this section, the results are presented.

### 4.1. High-Resolved Thermal Evaluation of Ex Vivo Myocardium Subjected to Radiofrequency Ablation

All the data provided by the FBGs were analyzed in MATLAB^®^ (Mathworks, Natick, MA, USA) environment. The ΔT was obtained with respect to the temperature recorded in the saline bath at the beginning of the RFA treatment (i.e., about 37 °C).

**Figure 5:**
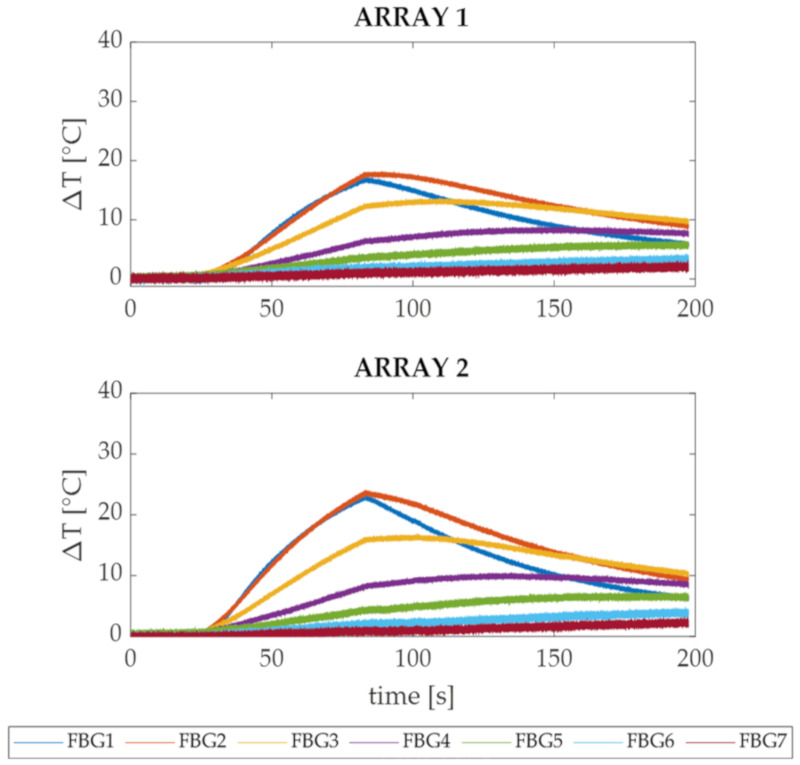
Temperature variations in time recorded by Arrays 1 and 2 during Trial 1 (RFA performed at 50 W). In the upper image, the seven trends relative to the FBGs housed in the Array 1 are shown, while in the bottom image the seven trends relative to the FBGs housed in the Array 2 are reported. The pre-treatment phase (i.e., 0 s–30 s), the RFA phase (i.e., 30 s–90 s) and the cooling phase (i.e., 90 s–200 s) are clearly distinguishable.

The temperature trends in time recorded by the 7 FBGs belonging to Array 1 and 2 during the RFA performed for 60 s with a delivery power of 50 W (Trial 1) are presented in Figure 5. All the ΔT trends show for both the arrays an immediate rise of temperature that started right after the application of the RF (i.e., 30 s on the time axis). The temperature increment is particularly visible for FBGs 1, 2 and 3, since they were subjected to a relevant temperature gradient, as were the closest sensors to the power delivery point.

After 60 s of treatment (i.e., about 90 s on the time axis), the energy delivery was interrupted and a cooling phase was registered mainly by FBGs 1, 2 and 3, while FBGs 4, 5, 6 and 7 kept recording a small and slow rise of temperature. For both Array 1 and Array 2, the most significant temperature changes were detected by FBGs 1 and 2, which were positioned 2 and 5 mm from the tissue surface, respectively. The maximum ΔT values recorded by Array 1 (see Figure 5, upper image) were 18 °C (i.e., detected by FBG 2) and 17 °C (i.e., detected by FBG 1), while for the Array 2 (see Figure 5, bottom image) they were 24 °C (i.e., detected by FBG 2) and 23 °C (i.e., detected by FBG 1). Higher ΔT values were measured by the FBGs belonging to Array 2 which was closer (i.e., 3.30 mm) to the RF antenna than the Array 1 (i.e., 6.30 mm).

**Figure 6:**
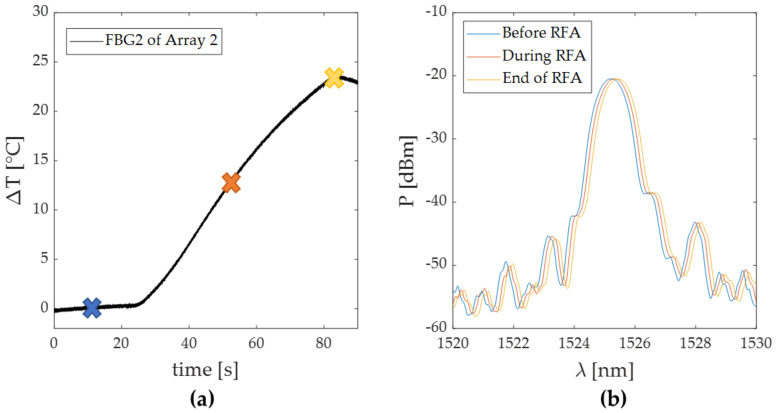
Focus on the temperature variation recorded during Trial 1 by FBG 2 of Array 2: (**a**) temperature trend zoomed in on the pre-treatment and RFA phases; (**b**) optical spectra relative to the FBG 2 recorded during the three different steps of the procedure marked as (**a**): before (blue line), during (red line) and at the end of the RF treatment (yellow line).

Of all sensors, FBG 2 belonging to Array 2 detected the highest temperature peak; in Figure 6a, a focus on the heating phase of its temperature trend is shown. Three colored marks have been reported on the temperature profile to denote three different steps of the RFA procedure: before the start of the RFA (i.e., blue cross), during the RFA (i.e., orange cross) and at the end of the RFA (i.e., yellow cross). In Figure 6b, the optical spectrum of the FBG 2 corresponding to the three different instants of time indicated in Figure 6a by the three crosses is shown, and the spectrum shift caused by the ΔT can be appreciated.

**Figure 7:**
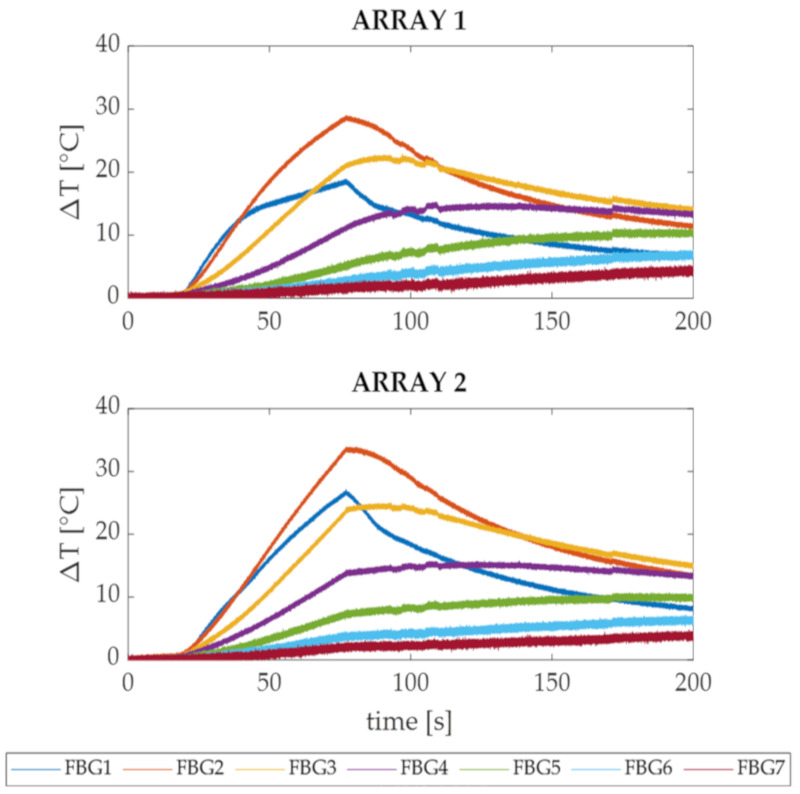
Temperature variations in time recorded by the Arrays 1 and 2 during Trial 2 (RFA performed at 60 W). In the upper image, the seven trends relative to the FBGs housed in Array 1 are shown, while in the bottom image the seven trends relative to the FBGs housed in Array 2 are reported. The pre-treatment phase (i.e., 0 s–25 s), the RFA phase (i.e., 25 s–85 s) and the cooling phase (i.e., 85 s–200 s) are clearly distinguishable.

The temperature trends in time recorded by the FBGs belonging to Arrays 1 and 2 during the RFA performed for 60 s with a delivery power of 60 W (Trial 2) are presented in Figure 7. Also in this case, the three treatment phases (i.e., before, during and after the RFA procedure) are clearly identifiable mainly from the trends recorded by FBGs 1, 2 and 3. Once again, FBGs 4, 5, 6 and 7 experienced lower ΔT values, slow temperature rises and subtle temperature increases even during the cooling phase. For both Array 1 and Array 2, the most significant temperature increments were detected by FBGs 1, 2 and 3. In Array 1, FBGs 2 and 3, which were placed 3 and 6 mm deep, recorded the maximum ΔT values of 29 °C and 22 °C, respectively. Otherwise, in Array 2, FBGs 1 and 2 which were placed directly under the surface (i.e., FBG 1) and at 3 mm from the surface (i.e., FBG 2), detected the maximum values of 27 °C and 33.5 °C, respectively. Again, Array 2, which was the closest to the RF antenna (i.e., 6.30 mm vs. 6.60 mm), was the one that reported the highest ΔT.

**Figure 8:**
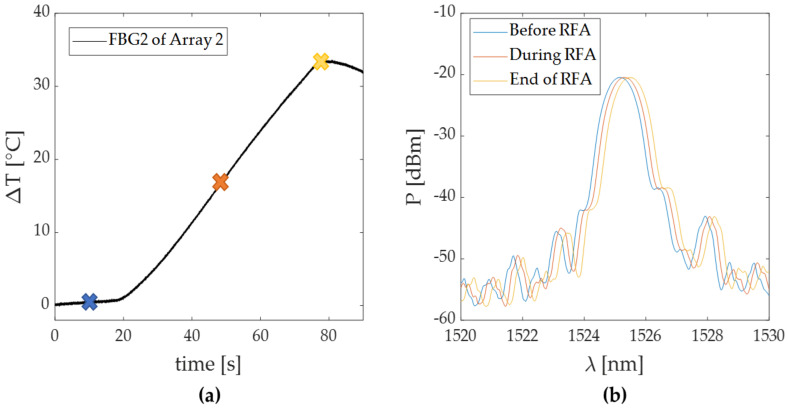
Focus on the temperature variation recorded during Trial 2 by FBG 2 of Array 2: (**a**) temperature trend zoomed on the pre-treatment and RFA phases; (**b**) optical spectra relative to the FBG 2 recorded during the three different steps of the procedure marked as (**a**): before (blue line), during (red line) and at the end of the RF treatment (yellow line).

In this trial, the highest temperature peak was detected by FBG 2 of Array 2. In Figure 8a, a focus on the heating phase of its ΔT trend is reported, with three colored marks once again, to show the aforementioned RFA phases. The FBG 2 reflected spectrum collected before, during and at the end of the treatment is reported in Figure 8b.

In the results reported in Figure 5, Figure 6, Figure 7 and Figure 8, the Δλ_B_ experienced by the sensors has been considered caused by the ΔT. This was confirmed by an experiment which showed an RF procedure on myocardial tissue followed by a complete cooling phase.

**Figure 9:**
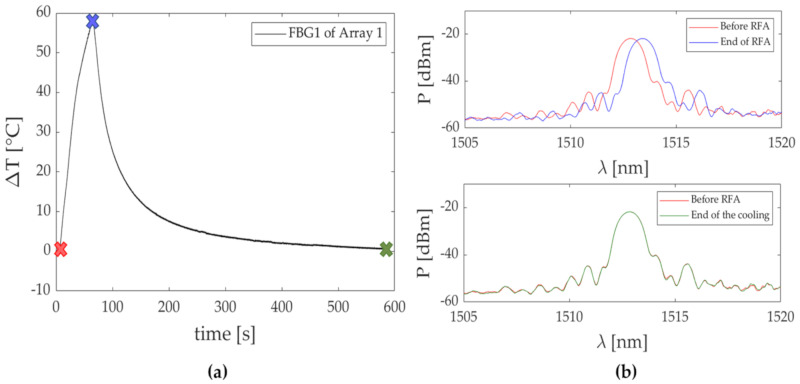
(**a**) Temperature trend recorded by FBG 1 embedded in Array 1 during an RF procedure on myocardial tissue; (**b**) optical spectra relative to FBG 1 recorded during the three different steps of the procedure marked as (**a**). In the upper image, the spectra collected before (red line) and at the end of the RF procedure (blue line). In the bottom image, the spectra collected before the RFA (red line) and at the end of the cooling phase (green line).

Figure 9a reports the temperature increment of one of the FBGs (i.e., FBG 1) embedded in the Array 1 during the mentioned experiment. 

Once again, three colored marks were reported to identify three moments of the procedure: before the start of the RFA (i.e., red cross), end of the RFA (i.e., blue cross) and at the end of the cooling phase (i.e., green cross). Figure 9b shows the behavior of the optical spectrum of the FBG 1 corresponding to the three instants of the procedure. Figure 9b (upper image) shows the shift experienced by the spectrum toward higher wavelengths from the start of the RFA to the end of the energy delivery (red and blue lines, respectively). Furthermore, the spectrum returns to the starting point at the end of the cooling (see Figure 9b, bottom image). This phenomenon is visible from the overlapping of the two spectra collected before the RFA and at the end of the cooling (red and green lines, respectively). Such behavior suggests that the residual strain can be considered negligible.

### 4.2. Visual Evaluation of Ex Vivo Myocardium Subjected to Radiofrequency Ablation

**Figure 10:**
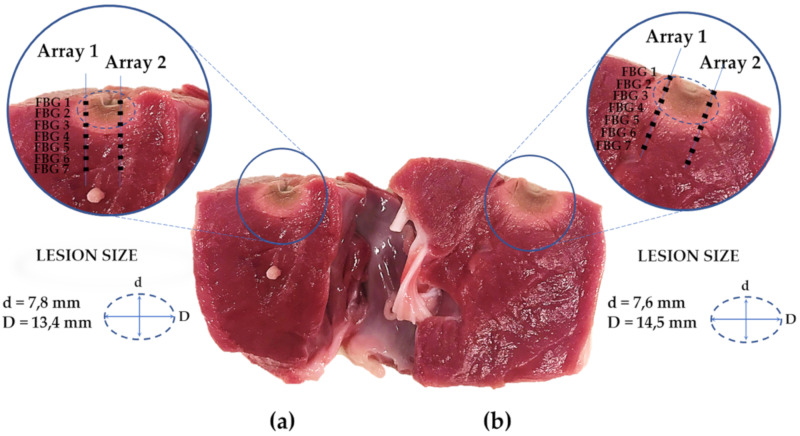
Picture of the two half-sections of the treated tissues sliced in correspondence to the lesions and focused on the positioning of the sensors within the specimens: (**a**) section of myocardium subjected to RFA with power setting of 50 W (Trial 1) with a zoom-in on Lesion 1; (**b**) section of myocardium subjected to RFA with power setting of 60 W (Trial 2) with a zoom-in on Lesion 2.

At the end of the RFA treatments, the myocardial specimens were sliced in correspondence to the RF antenna tip placement and divided into two halves. Shapes and dimensions of the produced lesions are shown in Figure 10, along with a zoom-in which clarifies the FBGs’ positions in the tissue with respect to the damaged tissue and antenna placement.

The damages presented elliptic shapes. The dimensions were evaluated manually by means of a digital caliper. The lesion relative to the Trial 1 (Lesion 1) was 7.8 mm long and 13.4 mm wide, while the one relative to the Trial 2 (Lesion 2) presented a length of 7.6 mm and a width of 14.5 mm. In both the experimental configurations, the first three FBGs of all arrays were contained within the portion of the thermally damaged tissue.

## 5. Discussion

RFA is the most commonly used procedure in cardiac ablation routines [2,3]. The dimensions of the produced lesions, and in turn, the good outcomes of such procedures, deeply depend on the temperature values reached within the treated tissues. Thus, being able to know the temperature distribution into the myocardium during RFA can be beneficial to predict the size of the lesion produced. FBG sensors are well suited to this purpose, as they permit high-resolved and distributed temperature measurements, thanks to their features (i.e., small dimension, flexibility, multiplexing capability and immunity to electromagnetic fields [23,24]).

In this study, the feasibility assessment of FBGs in performing millimetric-scale, multi-point temperature measurements within ex vivo myocardial tissue undergoing RFA is presented. Two fiber optic arrays containing 7 FBGs each (for a total number of 14 measurements sites) were used. Firstly, the response to temperature of the 14 sensors was evaluated showing S_T_ values of about 0.01 nm·°C^−1^. The two arrays were than positioned into two myocardial specimens at different distances from the RF antenna (see Figure 4). Two experimental trials were performed at 50 W (Trial 1) and 60 W (Trial 2) for 60 s of treatment time.

In both the experiments, the ΔT trends in time of the two arrays presented similar profiles, among which the three treatment phases are clearly distinguishable: before, during and after the energy delivery (Figure 5 and Figure 7). Nevertheless, during the cooling phases of Trial 1 and Trial 2, it is possible to observe that the first three FBGs showed a visible decrease in temperature, and FBGs 4, 5, 6 and 7 show a slow ΔT rise followed by an equally slow descent. This can be explained by taking into consideration the mechanism of heat transfer applied to biological tissues [28]: in fact, while the areas closest to the antenna are immediately heated due to the high thermal gradient, the surrounding areas suffer a delay caused by the heat diffusion from the area at high temperature (close to the antenna) to the peripheral one (far from the antenna).

FBGs 1 of each array were employed as reference positions for the placement of the other sensors and have been used to verify the influence of the cooling effect caused by the surrounding environment on different distances from the specimen surface. As expected, in Trial 1 the temperature values measured by FBGs 1 and 2 are almost comparable (see Figure 5), while in Trial 2 FBGs 1 measured a significantly lower ΔT (see Figure 7) with respect to FBGs 2. In fact, since in Trial 1 the FBGs 1 were 2 mm deep in the tissue, they were less affected by the thermal cooling compared to those in Trial 2. On the contrary, in Trial 2 FBGs 1 were placed right under the surface, so the cooling effect caused by the saline solution at about 37 °C deeply influenced their temperature trends, showing lower ΔT than those detected by FBGs 2. Instead, the FBGs 2 that were placed 5 mm (in Trial 1) and 3 mm (in Trial 2) under the surface were minimally affected by the cooling influence.

Starting from FBG 2 and proceeding deeper into the tissue up to FBG 7, the experimental data present even lower ΔT values for both the trials (see Figure 5 and Figure 7). It worth noting that, although the distance between two consecutive sensors is minimal (i.e., 2 mm), the gap between the measured temperatures is relevant. For example, during both Trial 1 and Trial 2, the FBG 2 belonging to Array 2 detected temperature values up to 8 °C higher than FBG 3, although their positioning within the specimen differed by only 2 mm in depth. Additionally, Array 2 measured a maximum temperature excursion of 19 ° C within a sensitive length of 9 mm (i.e., from FBG 2 to FBG 5) during Trial 1, while in Trial 2 the detected temperature excursion was 26 °C within the same sensitive length. Therefore, the large thermal gradient detected in such a small area of myocardial tissue points out the utility of a multi-point and high-resolved temperature monitoring strategy such as the FBG-based one.

The maximum values of temperature were recorded at the end of the discharge by the FBGs 2 of both the arrays: ΔT values of 18 °C and 24 °C were detected during Trial 1, while 29 °C and 33.5 °C were detected during Trial 2 by FBGs 2 belonging to Array 1 and Array 2, respectively. As is visible, in both the trials the higher temperatures were detected by the FBGs held in the array closest to the RF antenna. The temperature profiles of the FBG (i.e., FBG 2 held in Array 2 in both the tests) that recorded the highest temperature increments in Trial 1 and 2 are shown in Figure 6a and Figure 8a, respectively. Moreover, the corresponding optical spectra are presented for three different instants of time (before, during and after the RFA) in Figure 6b and Figure 8b. The Δλ_B_ produced by the thermal variation can be here appreciated. It worth noting that the spectrum shifts proportionally with the ΔT increase. The maintenance of the peak in time and the preservation of the spectrum shape suggest that the FBGs experienced no strain deformation during the entire duration of the treatment. Additionally, the absence of residual strain was confirmed by the overlapping of the two spectra (shown in Figure 9b) acquired before the RF treatment and at the end of an entire cooling phase.

In Figure 10, the lesions produced on the treated tissues are shown. As also reported in previous studies [11,29], the damaged areas exhibited elliptical shapes. The lesion produced in the trial performed at higher power is reasonably larger than the other one, thereby confirming that the sizes of the damaged areas increase proportionally with power value [11]. However, while the lesions can be compared in length (7.8 mm for Lesion 1 vs. 7.6 mm for Lesion 2), their dimensions are not comparable in width (13.4 mm for Lesion 1 vs. 14.5 mm for Lesion 2). Such results suggest that the lesion produced reaches a plateau in its length size for a power delivery of 50 W. This implies that for such power discharge values, heat diffusion in myocardial tissue may occur mainly width-wise rather than depth-wise. Zoom-ins on both the lesions are shown and the positions of the arrays with respect to the antenna placement and the ablated zone are specified. Considering that the specimens were immersed in a saline bath at about 37 °C, it is worth noting that the FBGs included into the ablated areas (i.e., the dark areas highlighted by the ellipses in Figure 10) were the ones which recorded ΔT values greater than 15 °C (i.e., the first three FBGs of both the arrays, for both Trial 1 and Trial 2). In fact, as is well known in the literature [4], irreversible damage occurs when myocardial tissues experience temperatures equal or higher than 50 °C.

To date, the detection of temperature trends of the electrode-tissue interface in cardiac micro-ablation routines and in ex vivo experiments is mainly performed by means of thermocouples and thermistors embedded into the delivery antenna [12,13,14,15,16]. In clinical practice, the use of this solution permits the avoidance of life-threatening complications, such as myocardial perforation or steam pops. Nevertheless, such a thermometric approach does not allow temperature measurements in the whole area subjected to ablation, but only in its superficial part. Moreover, the recorded temperature values are generally underestimated since such systems are affected by the cooling effect caused by the surrounding bodily environment. On the contrary, the presented FBG-based system offers the possibility of detecting the thermal evolution of several points within deep-seated tissue, and not only in the superficial zone. Furthermore, the high thermal sensitivity value (i.e., 0.013 nm·°C^−1^) and the small size of the sensors (i.e., 1 mm) allow extremely accurate and resolved measurements. For instance, in the present study the thermocouple embedded into the RF antenna tip measured superficial temperature values of 42 °C and 52 °C for Trial 1 and Trial 2, respectively, at the end of the power discharge. On the contrary, the FBGs held into the two arrays recorded the evolution in time of the intra-tissue temperature, reaching values of temperature variations of up to 33.5 °C (i.e., Trial 2, FBG 2 belonging to Array 2) for an overall tissue temperature of 70.5 °C at 2 mm in depth (that is about 15 °C higher compared to the one detected by the thermocouple).

Among contactless techniques, only MRI- [17,18] and ultrasound-based [30] solutions have been applied for myocardial temperature mapping during RF procedures. Despite the noticeable advantages given by the use of contactless technologies, they present evident limitations. In fact, the images provided by MRI technology are poorly accurate, not real-time and mainly affected by motion artifacts due to the heart beating movements. Moreover, the state changes occurring in the myocardium when temperatures higher than 50 °C are reached make ultrasound-based thermal maps unreliable. Instead, the use of arrays of FBGs allows accurate real-time measurements also at extremely high temperatures (i.e., above 100 °C [31]), with no motion artifacts.

## 6. Conclusions

In conclusion, this study presents the first attempt to assess the feasibility of FBGs in performing real-time, high-resolved temperature measurements within ex vivo myocardial tissue undergoing RFA. This study lays the foundations for the design of an FBG-based system for the detection of temperature trends that could allow investigating the main factors that influence the effects of RFA, with the aim of making RF cardiac ablation procedures ever safer and more controllable.

FBGs present several features that make their use optimal for ex vivo investigation: the arrays are bioinert, their small size and high flexibility allow easy insertion into the tissue, the immunity to electromagnetic fields permits MR- and CT-guided positioning, and their excellent metrological characteristics allow accurate temperature measurements with high spatial resolution.

Future studies will increase the number of experiments, increasing both the number of the measurement sites and the treatment settings (i.e., supplied power, treatment time, force of contact and irrigation flow of the catheter). This investigation aims at creating 3D thermal maps to establish a new understanding of how the abovementioned factors influence the RF procedure’s effects on the tissue. These new findings may be translated to the clinics to optimize the treatment settings and improve the clinical results.

## Figures and Tables

**Table 1:** The S_T_ values of the FBGs embedded in Array 1 and Array 2 calculated as the slope of the best fitting line.

	S_T_ [nm·°C^−1^]	
# FBGs	Array 1	Array 2
FBG 1	0.0098	0.010
FBG 2	0.0097	0.010
FBG 3	0.0098	0.010
FBG 4	0.0098	0.0099
FBG 5	0.010	0.010
FBG 6	0.010	0.010
FBG 7	0.0099	0.010

**Table 2:** Coordinates of the FBGs embedded in Arrays 1 and 2 for the configurations referred to the 50 and 60 W experiments. The tip of the RF antenna is considered to be the origin of the axes.

	Trial 1 at 50 W		Trial 2 at 60 W	
Array 1	x [mm]	y [mm]	x [mm]	y [mm]
FBG 1	−6.30	−2	−6.50	0
FBG 2	−6.30	−5	−6.50	−3
FBG 3	−6.30	−8	−6.50	−6
FBG 4	−6.30	−11	−6.50	−9
FBG 5	−6.30	−14	−6.50	−12
FBG 6	−6.30	−17	−6.50	−15
FBG 7	−6.30	−20	−6.50	−18
**Array 2**	**x [mm]**	**y [mm]**	**x [mm]**	**y [mm]**
FBG 1	3.30	−2	6.30	0
FBG 2	3.30	−5	6.30	−3
FBG 3	3.30	−8	6.30	−6
FBG 4	3.30	−11	6.30	−9
FBG 5	3.30	−14	6.30	−12
FBG 6	3.30	−17	6.30	−15
FBG 7	3.30	−20	6.30	−18
